# Imbalance between Emotionally Negative and Positive Life Events Retrieval and the Associated Asymmetry of Brain Activity

**DOI:** 10.3390/bs10010018

**Published:** 2019-12-30

**Authors:** Olga Razumnikova, Ekaterina Khoroshavtseva

**Affiliations:** Department of Psychology and Pedagogic, Novosibirsk State Technical University, Novosibirsk 630073, Russia; horoshavcevakatya@gmail.com

**Keywords:** emotion, autobiographical memory, electroencephalography, cortical areas, brain oscillations

## Abstract

Sustained focusing on a negative assessment of life events can create negative background and changes in the emotional feedback to new information. In this regard, it is important to assess the balance between self-assessment of emotional memories and their reflection in brain activity. The study was aimed at exploring the brain activity using electroencephalographic (EEG) analysis in six frequency ranges from delta to beta2 during the retrieval of positive or negative emotional memory compared with the resting state. According to ANOVA results, the most informative for differentiation of emotions were the alpha2 and beta2 rhythms with greater synchronization effect for positive than for negative emotions. The memory retrieval, regardless of the valence of emotions, was accompanied by alpha1 desynchronization at the posterior cortex. Self-assessment of the memory intensity was not significantly different due to emotion valences. However, the scores of positive emotions were related positively with beta2 oscillations at the left anterior temporal site, whereas for negative emotions, at the right one. Thus, the emotional autobiographical memory is reflected by activation processes in the visual cortex and areas associated with multimodal information processing, whereas differentiation of the valence of emotions is presented by the high-frequency oscillations at the temporal cortex areas.

## 1. Introduction

Memories induce vivid emotional experiences in their own right and influence perception of the external world depending on the emotional significance of the information. Studying the emotional impact of memories may help to understand their basic mechanisms and their disruption in psychiatric disorders [1]. Sustained focusing on a negative assessment of life events causes negative background while information processes and changes in the emotional regulation of many cognitive functions, thus facilitating the development of depression. In this regard, it is important to assess the balance between self-assessment of the vividness of emotional autobiographical memory and its reflection in brain activity.

Left and right frontal cortical areas have shown to be differentially involved in the regulation of the emotional states and of emotion processing e.g., [2,3,4]. The opposite effects of associations between positive emotions and a dominance of the left frontal electroencephalographic (EEG) activity, and of the negative affective states with more right hemispheric activity, are in line with both the valence–arousal model [5] or frontal EEG asymmetry model [3]. The frontal asymmetry in resting condition is considered a characteristic of affective flexibility and can be predictive of future specificity of emotional behavior or psychopathology [6,7,8]. On the other hand, these asymmetries can be interpreted in terms of executive control (i.e., left-lateralized executive control would inhibit the processing of negative distractors, whereas right-lateralized control inhibits positive distractors [9]) as well as in terms of reallocation of attentional resources to subjectively significant stimuli presenting potential threat at baseline and under stress [10,11]. 

Not only frontal EEG asymmetry but also parietal brain asymmetry seems to be assumed to reflect cortical arousal components related to affective states [5]. According to locationist hypotheses, there are single brain regions which are specifically associated with different emotion categories. However, a meta-analytic review of the human neuroimaging literature on emotion shows evidence consistent with a psychological constructionist approach, that is, widespread neural processing for the affective dimensions of valence and arousal but not specifically localized distinct brain regions are involved in neuronal operations of discrete emotion categories [12,13]. 

It is well known that alpha rhythms have frequently been related to affective processing as a result of reciprocal interactions between excitatory and inhibitory processing [14]. Alpha oscillations, especially originating from parieto-occipital regions, are related to the inhibitory process due to internally directed attention constituting mental states such as imagery [15]. However, other data do not support the suggested role of alpha oscillations in inhibition of distraction in working memory [16]. The controversial opinions may be caused by individual variability in total cortical activation due to not only the alpha but also other frequency distinct oscillations associated with differences in emotional regulation while performing cognitive tasks. For example, the extent of delta and theta synchronization during retrieval of autobiographical memories could be taken as a measure of emotional involvement and emotional arousal as the synchronization of these oscillations is stronger on presentation of emotional than neutral stimuli [17,18,19]. It was also shown that both the alpha and high-frequency oscillations may be useful to differentiate the emotional responses [19,20].

The review of affective characteristics modulating memory reveals emotional arousal and personal involvement in life events as two factors that have the largest impact on the vividness of autobiographical memory [21]. The standardized stimuli as pictures, scenes, and words do not fully approximate the emotional events and vivid recollections of everyday life. Greater understanding of individual emotion processing can be gained by using stimuli that are personally relevant and significant to the participant [22]. So, the aim of the study was to research the cerebral cortex activation using an analysis of not only alpha but also low-frequency delta and theta as well as high- frequency beta oscillations during mental memory retrieval of life events that had a positive or negative emotional character due to their self-assessment. We expected that the internally aimed retrieval of negative emotional episodes would be associated with increased right-hemispheric activation in the frontal and parietal areas involving both alpha and beta oscillations, whereas the retrieval of positive emotional memory would be accompanied by the synchronization effect with a dominance of the left-hemispheric activity.

## 2. Materials and Methods 

The sample consisted of 31 university students (22 female) ranging from 19 to 21 years of age (M = 20.1, SD = 0.87). All participants were right-handed according to the Annett Hand Preference Questionnaire and reported no neurological or psychiatric disorders. The study was approved by the ethics committee of the Department of Psychology and Pedagogic at the Novosibirsk State Technical University (UHREC Project 2015/04), and was conducted according to the Declaration of Helsinki. Before the beginning of the experiment, participants were acquainted with the experimental procedure and signed informed consent. 

For the EEG assessment, participants were seated in a comfortable chair with eyes closed in an acoustically shielded room. During the resting state and emotion-induced episodes, participants were instructed to close their eyes, sit quietly, and stay calm to avoid movement artifacts. Participants were asked to self-induce upon highly salient emotional memories related to negative (anger, sadness, etc.) (Sn) and positive (happiness, joy) (Sp) life events. After each part of the experiment, the participants were asked to evaluate the vividness of self-induced positive and negative emotion using the 7-point Likert scale, where 1 = ‘not at all’, 7 = ‘extremely’.

Recording and analyzing of the EEG was performed using Mitsar-201 software (Mitsar Products, Sankt-Peterburg, Russia) from 19-channel active AgCl electrodes mounted in an elastic cap according to the international 10/20 system during three-minute episodes at the rest (Sr) and the experimental situations: Sn and Sp. Signals were recorded at a sampling rate of 500 Hz and a bandwidth of 70 Hz. EEG signals were segmented into 2 s intervals. Segments with saccadic eye movements, eye blinks, muscular and other artifacts were removed using a visual inspection of EEG measures. In the analysis, 30 artifact-free segments of total duration 60 s per each subject for the control and each task of self-induced emotional condition were subjected to fast Fourier transformation. A Hanning window with 50% overlap of each epoch was used to prevent spurious estimates of spectral power. EEG power was estimated in six sub-bands, including 1–4 Hz (delta), 4–7 Hz (theta), 7–10 Hz (alpha1), 10–13 Hz (alpha2), 13–20 Hz (beta1), and 20–30 Hz (beta2). Finally, the power scores were log-transformed. 

Analysis of Variance (ANOVA) was realized to determine whether the power of EEG oscillations of (i) different frequency and (ii) cortical areas were significantly different during self-retrieval of positive, negative, and neutral emotional states. When the sphericity assumption was violated, degrees of freedom were computed applying Greenhouse–Geisser adjustments to the degrees of freedom. Effect sizes were also estimated using partial squared eta (η2) coefficients. All post hoc pairwise comparisons were performed using Tukey’s test. 

## 3. Results

### 3.1. Behavioral Data

Rating of the emotionally positive memory (M = 5.5, SD = 0.3) and negatively valenced retrieval (*M* = −5.3, SD * *=* * 0.2) did not discriminate significantly between affective categories (*p*  <  0.34). 

### 3.2. EEG Data: Frequency Specificity

Two-way repeated ANOVA on the mean spectral power for the six selected frequency ranges (Δ, θ, α1, α2, β1, and β2) and three experimental conditions (Sr, Sp, and Sn) revealed significant OSCILLATION × EMOTION interaction (F (10, 290) = 4.08, p = 0.00003; η^2^_p_ = 0.123), showing that the spectral power changes across memory retrieval contributed to theta1, alpha1, and beta1,2 oscillations. According to Tukey’s post hoc tests, both Sp, and Sn were associated with a power decrease in the low θ, α1 frequency bands and a power increase in the high β1 and β2 frequency bands (*p* < 0.0001) vs. Sr. Significant differences between emotional state-induced EEG oscillations are shown in Table 1. Post hoc test showed that there was a significant difference between positive and negative emotions in β2 oscillations (*p* < 0.002) with higher synchronization in Sp than Sn. According to planned comparison, Sp was accompanied by the α1 desynchronization and the α2 synchronization as compared with both Sr (F (1, 29) = 9.06, *p* = 0.005) and Sn (F (1, 29) = 4.79, *p* = 0.04). 

### 3.3. EEG Data: Regional Specificity

In the second step of analysis, the ANOVAs were realized for EEG power at 19 electrode sites according to the revealed frequency specificity. In the theta band, a significant EMOTION x SITE interaction was found (Table 2). Post hoc Tukey’s test showed the significant desynchronization effect at the Cz and Pz sites only for Sp (*p* < 0.01). The EMOTION × SITE interaction tended to be significant for both the alpha1 and beta 1 bands. Post hoc analysis revealed regionally similar alpha1 desynchronization at the posterior cortex during Sp and Sn as compared to Sr (*p* < 0.0001) together with the left-hemispheric effect in Sn vs. Sr and more pronounced right-hemispheric effect in Sp vs. Sr (see Table 2). Beta1 synchronization in Sp was higher at anterior temporal cortex as compared to Sr and Sn. In the beta2 band, EMOTION × SITE interaction was significant, reflecting significant emotion effects at the anterior cortex during Sp and at the left occipital site during Sn (see Table 2). 

Analysis of emotion-induced multidirectional effects of the alpha1 and alpha2 oscillations (i.e., α1 desynchronization and the α2 synchronization) indicated more pronounced differences between Sp and Sn at the F4, F8, Fz, and Cz sites (0.001 < *p* < 0.01). 

**Figure 1:**
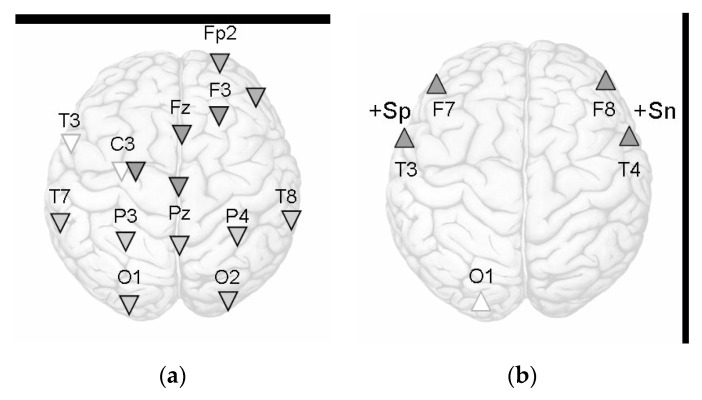
Emotion-induced changes in the alpha1 (**a**) and beta2 (**b**) oscillations. Note: Triangles down indicate a decrease of the alpha1power, triangles upward indicate the beta2 rhythm increase as compared to the rest (Sr): light triangles for Sn, dark triangles for Sp, light gray—common changes for both Sp and Sn; +Sp, +Sn indicate significant correlation between the beta2 power and self-assessment of emotion vividness.

Finally, we investigated an association between a rating of self-induced emotion and EEG. According to the Spearmen correlation test, a positive relationship between self-assessment of positive emotion and the power of beta2 oscillations at the T3 site (Rs = 0.37, *p* < 0.05) was found. There was also a positive relationship between the self-assessment of negative emotion and the beta2 oscillations at the T4 site (Rs = 0.36, *p* < 0.05). Self-assessment of negative emotion also tended to negative correlations with the alpha2 oscillations at the C3 and P3 sites during Sr (−0.30 < Rs < −0.34, 0.07 < *p* < 0.11). Integrally, the regional specificity of emotion-induced cortex activation for the alpha1 and beta 2 frequency bands is presented schematically by Figure 1.

In summary, the results hint at a broader association of emotional content of memory and brain activation represented by not only the alpha but also high-frequency beta oscillations. The significant differences between the emotions categories (Sp vs. Sn) are apparent in the alpha2 and beta2 oscillations. In the low theta and alpha1 bands, memory retrieval of emotional life events induces increased brain activation at the posterior cortex during both Sp and Sn in comparison to Sr. The Sn specificity is associated with the alpha1 desynchronization at the left temporal and central regions, and the Sp with the activation at the right anterior cortex. 

## 4. Discussion

While EEG recorded, the participants used autobiographical memory to retrieve positive or negative emotional events from real life. Autobiographical memory includes a complex set of different operations, namely episodic memory, self-reflection, emotion, visual imagery, executive functions, and semantic processes. A meta-analysis of the functional neuroanatomy of autobiographical memory gives evidence of a left-lateralized network, including select regions in the frontal, temporal, and posterior cortices [23]. It was found that emotional characteristics of episodic memory alter patterns of brain activity by additional recruitment of the right-hemispheric brain regions and emotion-specific structures in emotional re-experiencing [23,24]. So, obtained widespread changes of cortical oscillations are by the heterogenic nature of retrieval of life events with emotional content. 

The reconstructive characteristic of episodic memory, which includes the perceptual and conceptual elements [25], allows for different forms of autobiographical remembering by constructing memory representations with different combinations of details. According to the experimental paradigm, the participants could use different strategies when episode recalling: both verbal memory and visual images. Detected alpha desynchronization at the posterior cortical areas specialized in processing visual information (occipital cortex) and its multimodal characteristics (parietal regions) apparently reflects the active presentation of emotionally colored life episodes. 

The effect of regionally specific brain activation corresponds to the results of the emotional memory study, which showed that both the degree of such activation and its lateral features depend on the individual characteristics of memory strategies [26] and the psychometrically assessed self-confidence in the choice of behavior style [27]. EEG asymmetries are modulated by both the induction of certain emotions and individual affective dispositions [2]. Therefore, in the future, it is necessary to determine which personality traits (for example, a disposition to use approach/avoidance strategies, emotional intelligence, or neuroticism) make the most significant contribution to this effect of individual variability in EEG correlates of emotional autobiographical memory. Indeed, it was found that attachment representations differentially affect the parietal organization of hemispheric asymmetry during the retrieval of emotional memories [27]. 

Both valence and arousal represent two dimensions in which the neural activation deriving from emotional induction can be differentiated. The obtained results reveal that the most informative for the differentiation of emotion valence are the alpha2 and beta2 oscillations with greater synchronization of the high-frequency biopotentials during Sp than during Sn. It indicates the higher brain activation due to the search for the behavioral reactions required to avoid such negative situations. This effect of greater demand for brain activation when experiencing negative emotions is emphasized by multidirectional changes in the alpha 2 rhythm reactivity with synchronization of the oscillations during Sp but desynchronization during Sn. Bilateral beta2 synchronization in the anterior cortex is in line with the findings and shows a similar effect during internal induction emotional states [28]. 

The weak relationship between self-assessment of emotion vividness and EEG reactivity can reflect an imbalance between the subjective view on emotional experience and brain activation induced by a strategy of episodic memory retrieval. Only beta2 synchronization in the temporal cortex is associated with self-assessment score according to the valence–arousal model [5]: positive emotion correlates with the biopotentials at the T3, and negative with the beta2 power at the T4. Another reason for the imbalance between the subjective scores of emotions and their reflection in brain activity can be a positive mood shift during the experiment, which can weaken the emotional differences in the retrieval of affective states. As mentioned above, the intense variability in personality emotional experience and emotion induction [2,26,27] can also be the reason for the lack of significant EEG correlates of self-rating of emotional vividness.

## 5. Conclusions

The brightness of emotional autobiographical memory is reflected by activation processes in the visual cortex and areas associated with multimodal information processing, when the valence of internally induced emotions is differentiated by the neural systems of the temporal cortex areas. The activation effect is presented by the desynchronization of the low-frequency theta and alpha oscillations as well as by synchronization of the high-frequency beta rhythms. Self-assessment of the vividness of the emotional memory weakly correlates with EEG during retrieval of emotional life events, which indicates an imbalance between subjective scores of affective states and their reflection in brain activity. 

## Figures and Tables

**Table 1:** Significant differences between emotional state-induced EEG oscillations.

Frequency Band	Neutral Emotion (Sr)	Positive Emotion (Sp)	Negative Emotion (Sn)
Theta	0.689 (0.049)	0.659 (0.039) *	0.668 (0.040) *
Alpha1	0.940 (0.094)	**0.848** (0.086) *	0.873 (0.087) *
Alpha2	0.936 (0.059)	**0.965** (0.068)	0.918 (0.069)
Beta1	0.473 (0.046)	0.510 (0.048) *	0.488 (0.049) *
Beta2	0.252 (0.038)	0.345 (0.043) *	0.301(0.043) *^,^#

Note. * marks significant differences in Sp and Sn vs. Sr, #—Sp vs. Sn, bold—Sp in α1 vs. α2 compared with Sr and Sn.

**Table 2:** ANOVA results indicated regional effects of EEG differences between emotional states.

Frequency Band	F (df 36,1044)	*p*	η^2^_p_	Emotional States	Sites of Effects
Theta	1.94	0.04	0.06	Sp vs. Sr	Cz, Pz
Alpha1	1.63	0.12	0.05	Sn vs. Sr	T3, C3 and T5, T6, P3, Pz, P4, O1, O2
				Sp vs. Sr	Fp2, F4, F8, Fz, Cz, C3 and T5, T6, P3, Pz, P4, O1, O2

Beta1	1.90	0.06	0.06	Sp vs. Sr, Sn	T3, T4
Beta2	3.28	0.003	0.10	Sp vs. Sr	F7, F8, T3, T4
				Sp vs. Sn	T3, T4
				Sn vs. Sr	O1

Note. Sp, Sn, and Sr—experimental states of positive, negative, and neutral emotional states, respectively.
